# Adaptable bioinspired special wetting surface for multifunctional oil/water separation

**DOI:** 10.1038/srep39970

**Published:** 2017-01-04

**Authors:** Maryna N. Kavalenka, Felix Vüllers, Jana Kumberg, Claudia Zeiger, Vanessa Trouillet, Sebastian Stein, Tanzila T. Ava, Chunyan Li, Matthias Worgull, Hendrik Hölscher

**Affiliations:** 1 Institute of Microstructure Technology (IMT), Karlsruhe Institute of Technology (KIT), Hermann-von-Helmholtz Platz 1, 76344 Eggenstein-Leopoldshafen, Germany; 2 Institute for Applied Materials (IAM) and Karlsruhe Nano Micro Facility (KNMF), KIT, Hermann-von-Helmholtz Platz 1, 76344 Eggenstein-Leopoldshafen, Germany

## Abstract

Inspired by the multifunctionality of biological surfaces necessary for the survival of an organism in its specific environment, we developed an artificial special wetting nanofur surface which can be adapted to perform different functionalities necessary to efficiently separate oil and water for cleaning accidental oil spills or separating industrial oily wastewater. Initial superhydrophobic nanofur surface is fabricated using a hot pulling method, in which nano- and microhairs are drawn out of the polymer surface during separation from a heated sandblasted steel plate. By using a set of simple modification techniques, which include microperforation, plasma treatment and subsequent control of storage environment, we achieved selective separation of either water or oil, variable oil absorption and continuous gravity driven separation of oil/water mixtures by filtration. Furthermore, these functions can be performed using special wetting nanofur made from various thermoplastics, including biodegradable and recyclable polymers. Additionally, nanofur can be reused after washing it with organic solvents, thus, further helping to reduce the environmental impacts of oil/water separation processes.

Requirements of the developing world put a lot of strain on the planet, including environmental pollution and overuse of the resources. In recent decades accidental crude oil spills occurring during its transportation and extraction have resulted in devastating consequences for the environment, public health and economy^1^. During the infamous Deepwater Horizon oil spill in the Gulf of Mexico in 2010, caused by the explosion of the oil rig, 4.9 million barrels (780 million liters) of oil were released into the gulf waters^1^. The toxic crude oil devastated the coastal and marine ecosystems, and its long-term effects are still not fully understood^2^,^3^. The current technologies applied to oil spill cleaning include *in-situ* burning, adding chemical dispersants, and oily water collection followed by oil/water separation techniques such as floatation, coalescing and centrifuge^4^,^5^. Conventional techniques are limited by low efficiency, high costs and generation of secondary pollutants^6^. Besides oil spills, such industries as petrochemical, textile and metallurgy produce oily wastewater which has to be processed and separated according to strict specifications^6^.

The development of advanced materials with special wetting properties that can selectively remove water or oil during separation offers a great potential for handling future oil spill disasters and aiding the industries. Superhydrophobic materials are characterized by high apparent water contact angles (CA) above 150° and low roll-off angles, and their roughness-induced non-wettability is described by fundamental Wenzel and Cassie-Baxter models. They predict that superhydrophobicity can be achieved by adding roughness to hydrophobic surfaces (intrinsic CA > 90°), while adding it to the hydrophilic surfaces (CA < 90°) enhances wettability^7^,^8^. Understanding of the classical models has been greatly extended in the recent decades, and it was theoretically predicted and experimentally shown that surfaces non-wettable by water and low surface-tension liquids such as oils can be fabricated on materials with CA < 90° by controlling the local surface curvature of the roughness^8^,^9^. A term “superhygrophobic” (“hygro” is “liquid” in Greek) was suggested to describe superhydrophobic and superoleophiobic surfaces^10^. Moreover, based on theoretical and experimental results, the intrinsic CA = 65° was suggested as a boundary above which surface superhydrophobicity can be attained by adding nano- and microscale roughness^11^,^12^,^13^.

Selective separation of either oil or water from oil/water mixtures requires two different types of materials with special wetting properties: superhydrophobic/superoleophilic for oil removal, and underwater superoleophobic for water removal. Additionally, two material geometries exist to perform absorption or filtration of the selected fluid from the mixture: mesh-like porous structure as a filter, and sponge- or powder-like structure as an absorber^14^. As a result, multiple fabrication techniques and materials are used to realize seemingly similar oil/water separation tasks. For example, aerogels, powders, graphene oxide films, sponges modified with graphene, carbon nanotubes (CNT), nanoparticles or low-surface energy chemicals are used for oil absorption from water^4^,^15^,^16^,^17^,^18^,^19^,^20^,^21^,^22^,^23^,^24^; superhydrophobic CNT membranes, metal meshes or textiles chemically modified or coated with metal oxide nanoparticles and nanorods are utilized for oil filtration from oil/water mixtures^25^,^26^,^27^,^28^,^29^,^30^,^31^; and underwater superoleophobic porous nanotube membranes, meshes and textiles modified with palygorskite powders, zeolites, TiO_2_ nanostructures, nanoparticles, zwitterionic polymers or cellulose aerogels are used for filtrating water from oil/water mixtures^32^,^33^,^34^,^35^,^36^,^37^,^38^,^39^. To ensure a better control over the oil/water separation process, materials which switch their wetting properties in response to external stimuli such as changing electric or magnetic field, pH, temperature or light illumination, and pre-wetted materials were developed^6^,^40^,^41^,^42^,^43^,^44^.

Contrary to artificial surfaces, biological surfaces have evolved to perform various functions necessary for the survival of an organism in its specific environment. The top surface of the leaves of many floating plants is superhydrophobic in order to provide buoyant support on water and increase gas exchange through stomata pores, while the submerged bottom surface of the leaves is wettable by water^45^,^46^,^47^,^48^. For example, the Lotus leaf is superhydrophobic on top and underwater superoleophobic on the bottom side^48^. The surface wettability of the plant leaves is enhanced by a variety of nano- and microstructures such as hairs, waxes or cuticular folds^47^. Taking inspiration from the multifunctionality of plant surfaces into the global problem of oil/water separation, we developed an artificial surface, which by using a set of fabrication and modification techniques, can be adapted to perform different functionalities necessary for efficient oil/water separation: selective separation of water or oil by change of surface wettabilty; variable oil absorption capacity; continuous gravity driven separation of oil/water mixtures by filtration (Fig. 1). The presented techniques can be applied to a wide selection of polymeric materials, including biodegradable and recyclable polymers, and fabricated surface can be reused after washing with organic solvents. Bioinspired superhydrophobic nanofur surface is fabricated using a hot pulling method, in which nano- and microhairs are drawn out of the polymer surface due to adhesion to a heated sandblasted steel plate^49^,^50^. By combining the presented methods a custom oil/water separation platform in terms of material, filtration fluid and separation type can be created. For example, an oil absorber with high oil absorption capacity, a porous underwater superoleophobic membrane for water filtration or an oil filter, all made from the same biodegradable polymer can be realized.

## Results and Discussion

**Figure 2:**
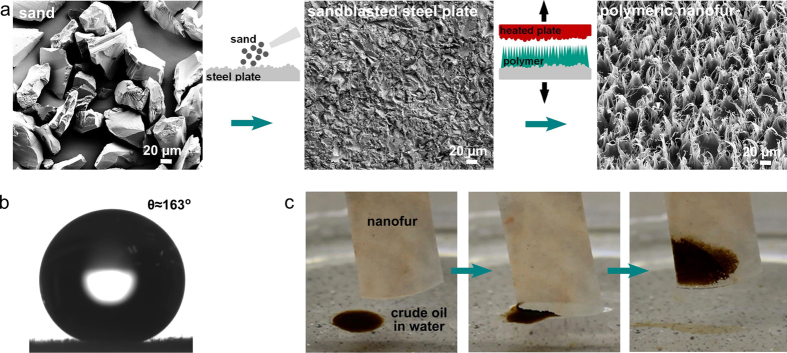
Nanofur fabrication procedure and wetting properties. (**a**) SEM and schematic images of the nanofur fabrication steps. Hot pulling with a heated sandblasted steel plate is used to produce nanofur. SEM images of the sand, sandblasted plate and polycarbonate (PC) nanofur surface are taken at the same magnification for better comparison. (**b**) Photograph of a water droplet on the superhydrophobic PC nanofur surface with contact angle *θ* ≈163°. (**c**) Crude oil spill cleanup by PC nanofur submerged into crude oil spill in a Petri dish. Nanofur absorbed most of the oil within few seconds.

Bioinspired nanofur is a polymeric surface covered by a dense layer of nano- and microhairs fabricated using a hot pulling technique schematically shown in Fig. 2a^49^,^50^,^51^. Hot pulling is a modified hot embossing process, in which demolding forces occurring during polymer and mold insert separation are used to fabricate high aspect ratio polymeric nano- and microstructures^52^,^53^,^54^. A steel plate sandblasted with rough aluminum silicate abrasive and then with finer aluminum oxide sand serves as a mold insert in hot pulling. The mold plate is heated above the glass transition temperature of the polymer, and pressed into the flat polymer foil attached to the bottom plate. Softened polymer fills the microcavities of the sandblasted mold, and the subsequent mold retraction creates a topography consisting of microcavities surrounded by dense nano- and microhairs on the polymer surface. Step-by-step nanofur fabrication procedure and corresponding scanning electron microscopy (SEM) images of the fine sand used for sandblasting, sandblasted steel plate and polycarbonate (PC) nanofur are shown in Fig. 2a.

The nanofur is superhydrophobic with typical contact angles above 150° (Fig. 2b), as its hierarchical nano- and microhairs entrap the air and support the water in Cassie-Baxter wetting state. The nanofur superhydrophobicity is achieved solely by the micro- and nanoscale roughness created during hot pulling. The superhydrophobic nanofur is superoleophilic, and can be used for selective oil/water separation^49^. The as-prepared nanofur successfully cleans an imitated crude oil spill (EK 651, MiRO refinery, Germany) when submerged into contaminated water by absorbing most of the oil within seconds, as shown in Fig. 2c. The oil remains locked in the nanofur hairs even after its removal from water.

### Biodegradable, recyclable and reusable nanofur

**Figure 3:**
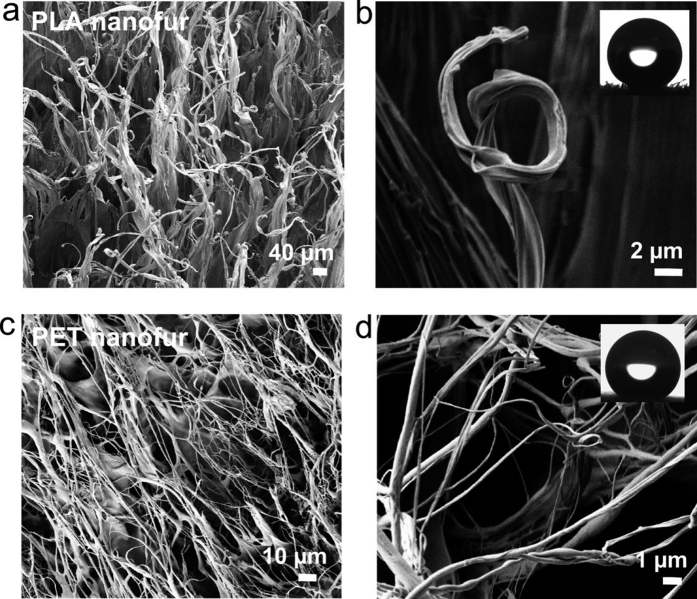
SEM images and wetting properties of biodegradable nanofur (polylactic acid (PLA)) and recyclable nanofur (polyethylene terephthalate (PET)). (**a**) PLA microhairs are hundreds of microns long. (**b**) Higher magnification SEM image reveals submicron structures on the hairs. Water contact angle (WCA) of 156° is in the inset. (**c**) PET nano- and microhairs form a mesh parallel to the surface. (**d**) The diameter of PET nanohairs is below 100 nm in some sections. WCA of 153° is in the inset.

Replacing conventional polymers derived from fossil fuels with biodegradable polymers from renewable resources is essential for reducing the environmental impact of many industries and households. Using biodegradable materials to handle such environmental disasters as crude oil spills is of an increased significance. Therefore, a biodegradable nanofur was fabricated from polylactic acid (PLA). PLA is a biodegradable polymer derived from corn starch or sugarcane^55^. To fabricate PLA nanofur for oil spill cleanup the hot pulling method was used in a velocity and force-controlled mode with the following parameters: *T* = 155 °C, embossing velocity 20 mm/min, embossing force 5.5 kN, retraction velocity 100 mm/min. The surface of the fabricated PLA nanofur is covered in high aspect ratio microhairs, as shown in SEM image in Fig. 3a. High magnification SEM image in Fig. 3b reveals submicron grooves on the PLA microhairs. The biodegradable nanofur is superhydrophobic (inset in Fig. 3b) and superoleophilic, and can be used for environmentally friendly oil/water separation.

Reusing recycled polymers for oil/water separation is another approach for reducing environmental impact of oil spills on the ecosystems and polymeric material accumulation in nature. To fabricate recyclable nanofur, we used films of polyethylene terephthalate (PET). PET is one of the most common fossil fuel-based polymers used for fabricating plastic bottles, textiles and packaging materials, and its recycling is widely established in the world. Using the hot pulling technique we fabricated recyclable PET nanofur. The parameters used for fabrication are: embossing temperature 275 °C, embossing velocity 10 mm/min, penetration depth 200 μm, retraction velocity 100 mm/min. SEM images of the PET nanofur are shown in Fig. 3c,d. PET nano- and microhairs have an average size of 470 ± 240 nm and minimal size below 100 nm. The recyclable nanofur is superhydrophobic, as can be seen in the inset in Fig. 3d. A unique characteristic of the PET nanofur topography compared to nanofur made from other polymers is that its hairs form a mesh parallel to the bulk polymer surface. Additionally, superhydrophobic/superoleophilic nanofur was successfully fabricated from such recyclable polymers as polypropylene (PP) and polyethylene (LDPE and HPDE).

Moreover, the oil-contaminated nanofur can be reused after washing it with organic solvents^4^,^56^. To investigate the nanofur reusability, polypropylene (PP) nanofur was contaminated with oil and subsequently cleaned with isopropanol (stirred twice at 200 rpm for 5 min, and dried), which removes the absorbed hydraulic oil. The oil absorption capacities of the original PP nanofur and nanofur after five absorption/washing cycles are, correspondingly, 310 ± 46 g/m^2^ and 288 ± 49 g/m^2^. Washing does not affect the wetting properties of the nanofur, as the water contact angles before and after five washing cycles are 151 ± 4°. Reusability, in addition to the possibility of nanofur fabrication from biodegradable and recyclable polymers, makes the nanofur especially attractive for large-scale environmentally friendly oil/water separation.

### Variable oil absorption

**Figure 4:**
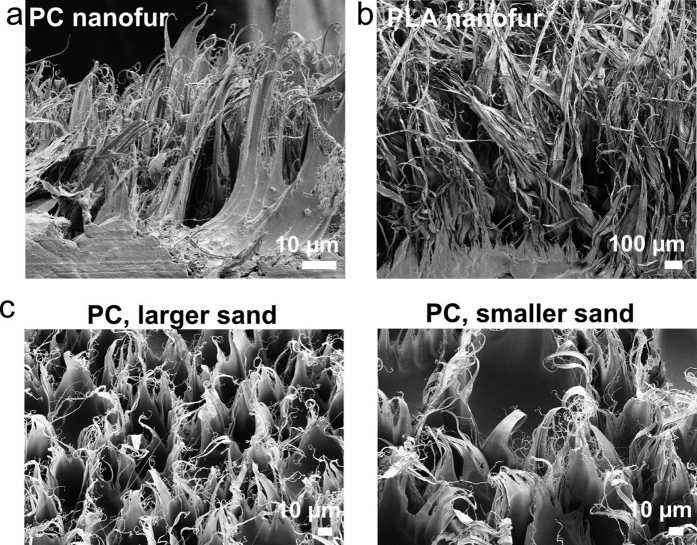
Oil absorption capacity depends on nanofur hair morphology. (**a**) Short-haired polycarbonate (PC) nanofur is capable of absorbing up to 127 g/m^2^ of oil. (**b**) Long-haired polylactic acid (PLA) nanofur oil absorption capacity is up to 479 g/m^2^. (**c**) SEM images of PC nanofur fabricated using molds sandblasted with different size sands (53 ± 3 μm, 17 ± 1 μm) are taken at the same magnification. The sand size influences the mold roughness and, thus, the demolding forces occurring during polymer and mold separation, resulting in different nanofur hair densities and lengths.

The oil absorption capacity of the hair-covered superhydrophobic/superoleophilic surface depends on the hair length and morphology, as was recently shown in a study of oil absorption by different superhydrophobic plants^57^. Another important functionality of the nanofur is the ability to vary its hair morphology by using different materials and fabrication parameters. Hot pulling is a versatile technique which can be applied to various thermoplastic polymers as described above. Different molecular structures and physical properties of the polymers used for hot pulling result in specific micro- and nanostructures of the pulled hairs unique for each polymer. Different hair morphologies can be seen in the cross-sectional SEM images of PC and PLA nanofur shown in Fig. 4a,b. The length of hairs varies from tens of microns in PC nanofur to millimeters in PLA nanofur. Such difference in length greatly affects oil absorption. Oil absorption capacity of the nanofur was measured using hydraulic oil (Total Azolla ZS 10), and for the short-haired PC nanofur is up to 127 g/m^2 ^49. In comparison, the long-haired PLA nanofur absorbs up to 479 g/m^2^ of oil.

The hair morphology of the polymeric nanofur can be further changed by varying the fabrication parameters such as embossing temperature and force, penetration depth, mold roughness, demolding temperature and velocity. Although a detailed analysis of the hair morphology over a wide parameter space is beyond the scope of this paper, one notable example is the effect of the mold roughness. The size of the sand used for mold sandblasting influences the roughness of the mold and, thus, the demolding forces occurring during polymer and mold separation, resulting in different nanofur hair densities and lengths, as shown in SEM images Fig. 4c.

### Selective separation: oil-removing and water-removing nanofur

**Figure 5:**
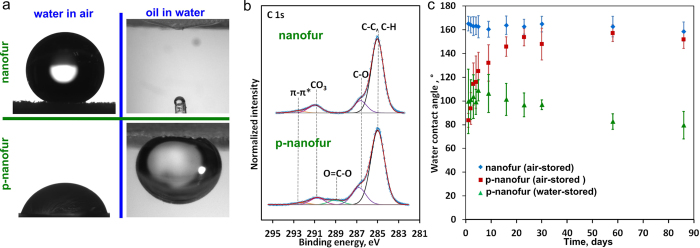
Controlling nanofur wetting properties using plasma treatment and p-nanofur hydrophobic recovery prevention. (**a**) Photographs of water droplets in air and oil droplets in water on untreated superhydrophobic/superoleophilic PC nanofur surface and plasma-treated hydrophilic/underwater superoleophobic p-nanofur reveal the change in wetting properties induced by the treatment. (**b**) The C 1s XPS spectra of PC nanofur and p-nanofur indicate incorporation of hydrophilic carboxyl functional groups on the p-nanofur surface. (**c**) Average water contact angles vs. storage time of untreated PC nanofur stored in air and plasma-treated p-nanofur stored in air and in water demonstrate that hydrophobic recovery of the p-nanofur can be prevented by storing in water. Error bars correspond to the standard deviation of at least 7 measurements.

Selective separation of either oil or water from the oil/water mixtures requires materials with opposite wetting properties. Most of the materials fabricated using existing technologies can separate only one of the two fluids, and separating the other one requires a different technology^15^,^16^,^17^,^18^,^19^,^20^,^21^,^22^,^23^,^24^,^25^,^26^,^27^,^28^,^29^,^30^,^31^,^32^,^33^,^34^,^35^,^36^,^37^,^38^. The possibility to selectively separate both fluids using the same material offers flexibility for different oil/water separation applications. The as-prepared polymeric nanofur is superhydrophobic/superoleophilic and can be used to remove oil from oil/water mixtures. To modify the nanofur functionality from oil-removing to water-removing, the surface energy of the as-prepared material is changed using plasma treatment. Plasma treatment is widely used to increase the surface energy of polymeric and other surfaces, and as a result increases their hydrophilicity^58^,^59^,^60^. Modification of the polycarbonate (PC) nanofur surface was carried out by argon plasma treatment (0.2 mbar, 30 W, 120 s). SEM images of the nanofur before and after plasma treatment did not reveal any changes in the surface topographies. The PC nanofur water contact angle (WCA) in air is 165 ± 6° on unmodified nanofur, and ranges from 50° to 133° on plasma-treated nanofur surface (p-nanofur) (left column in Fig. 5a). For comparison, WCA of the flat PC surface before plasma treatment is 88 ± 2°, and 52 ± 1° after. In water, the surface of the untreated nanofur is superoleophilic with underwater oil contact angle (OCA) close to 0°. However, plasma treatment of the nanofur results in underwater superoleophobicity of the surface (OCA > 150°), as can be observed in Fig. 5a (right column). The underwater superoleophobicity of the p-nanofur surface is a result of minimized contact between oil and solid due to water trapped in between the nano- and microhairs, similarly to air trapped on the untreated superhydrophobic nanofur surface^6^.

The surface chemistry of the as-prepared PC nanofur and nanofur treated with argon plasma was investigated by X-ray photoelectron spectroscopy (XPS). The core level C 1s XPS spectra of untreated and plasma-treated nanofur are shown in Fig. 5b. The C 1s envelope of the as-prepared PC nanofur can be decomposed into three peaks (285.0 eV peak corresponds to C-C and C-H bonds, 286.6 eV is due to C-O groups, and 291.0 eV is due to CO_3_)^61^. After Ar plasma treatment an additional peak at 289.0 eV representing O=C-O groups was obtained. The spectrum of plasma-treated sample indicates oxidation of carbon in atmospheric oxygen after plasma treatment. Incorporation of hydrophilic carboxyl functional groups on the surface renders the p-nanofur surface hydrophilic. The nanofur surface prior to plasma treatment contained 14.9% oxygen, compared to 24.6% oxygen at the p-nanofur surface after treatment.

Hydrophilization of the polymeric surfaces by plasma treatment necessary for separating water from oil/water mixtures is not permanent, which results in a so-called hydrophobic recovery or aging of the surface. Multiple mechanisms are responsible for the hydrophobic recovery, including reorientation of hydrophilic functional groups and diffusion^59^,^62^. However, the recovery rate can be greatly influenced by storage conditions, such as surrounding medium, temperature and humidity^59^,^60^,^62^. To study the hydrophobic recovery of the plasma-treated PC p-nanofur, we measured water contact angles of p-nanofur stored in ambient air and in deionized water for duration of 85 days, and compared them to contact angles of the as-prepared nanofur stored in air. The graph of average measured water contact angles as a function of storage time for these three groups of PC nanofur is shown in Fig. 5c. The results indicate the hydrophobic recovery of the p-nanofur stored in air, which recovers its high water contact angles (WCA = 154 ± 7°) approximately after three weeks of aging. The contact angles measured on p-nanofur stored in water, on the contrary, stay low. For example, the average WCA of p-nanofur samples is 100 ± 27° on the day of the treatment, and is 97 ± 4° after 30 days of storage in water. The consequent reduction of WCA of the p-nanofur stored in water for longer times could be attributed to water absorption by polycarbonate^59^. The results shown in Fig. 5c demonstrate that hydrophobic recovery of the p-nanofur can be prevented by storing it in water. These results are consistent with previously reported studies of hydrophobic recovery of plasma-treated polymer surfaces^59^,^60^,^63^. Therefore, the underwater superoleophobic properties of the p-nanofur, necessary for oil/water separation, can be retained by storing it in water. Nanofur and p-nanofur selectively remove oil or water, and cover the full range of oil/water separation applications which technical specifications require either one or the other fluid to be removed from the oil/water mixture.

### Porous nanofur for continuous oil/water filtration

**Figure 6:**
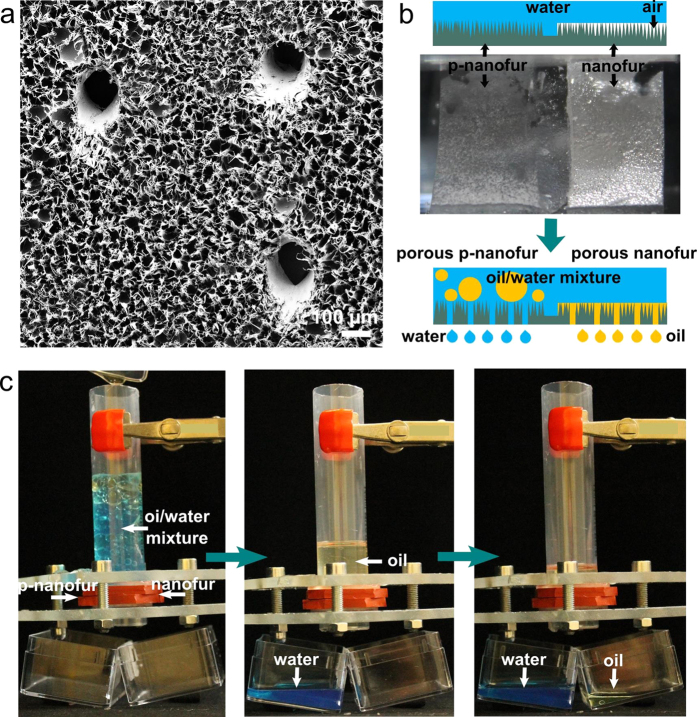
Porous nanofur membrane for continuous oil or water filtration from the oil/water mixture. (**a**) SEM image of porous nanofur fabricated by perforation with microneedles. (**b**) Schematic and optical image of combined side-by-side nanofur and p-nanofur underwater: air-retaining superhydrophobic nanofur is silvery due to light reflection from the air-water interface, and hydrophilic p-nanofur is opaque. Schematic of the filtration process by combined porous nanofur/p-nanofur filter: oil is drained though the pores in superhydrophobic/superoleophilic nanofur (water is retained above nanofur), and water is drained through the porous underwater superoleophobic p-nanofur. (**c**) Oil/water mixture separation by combined nanofur/p-nanofur filter: water (colored blue) penetrates through the p-nanofur (left), and cooking oil is filtered out through the nanofur (right).

Special wetting materials with pores allow continuous separation of both fluids from the oil/water mixtures by filtration. Porous materials are especially attractive for oil/water separation because of the wide use of porous membrane-based filtration technology in various industries. To extend the nanofur capabilities to filtration of oil/water mixtures, we fabricated micropores in the PC nanofur surface by perforating it with microneedles (160 μm in diameter). SEM image of the resulting porous nanofur membrane is shown in Fig. 6a. Due to opposite wetting properties of nanofur and p-nanofur, porous membranes made from these materials are capable of selectively removing either oil or water from the mixtures. The differences between the filtration processes characteristic to each material are highlighted in Fig. 6b. An optical image of side-by-side nanofur/p-nanofur submerged underwater indicates the superhydrophobicity of the untreated nanofur (silvery layer indicates air trapped by the hairs) and hydrophilicity of the p-nanofur (opaque in water). With addition of pores into the superhydrophobic/superoleophilic nanofur, the oil is absorbed on the surface after replacing the air (Fig. 2c), and is drained through the pores, while water is retained above the surface (Fig. 6b). The situation is reversed in case of porous underwater superoleophobic p-nanofur, where water is filtered out by the membrane and oil stays on top.

To demonstrate oil and water separation from the oil/water mixture by nanofur, we produced a filter by gluing together nanofur and p-nanofur into one membrane shown in optical image and schematic in Fig. 6b, and perforated 100 microholes on each side. The porous membrane was inserted into a filtration device and oil/water mixture was added on top of it (Fig. 6c). Water used in this experiment was colored with blue ink for better visibility, while oil was uncolored. In the images shown in Fig. 6c, it can be seen that water penetrates thorough the p-nanofur half of the porous filter and is collected below, and oil penetrates thorough the untreated nanofur. There is no oil/water mixture left on top of the device after the separation is finished. Due to amphiphilicity of the unstructured filter backside droplets can spread to the opposite part of the filter resulting in cross-contamination (no oil droplets are observed in the separated water, but small water droplets are sometimes seen in oil). The presented results demonstrate that porous nanofur/p-nanofur filter can separate water and oil from the mixture.

Using nanofur for oil/water separation by filtration, together with controlling filtration fluid, absorption and fabrication material will help to simplify various oil/water separation tasks and satisfy requirements imposed by the specific operational conditions of industrial and environmental separations. For example, due to the variety of polymers available for nanofur fabrication, the oil/water separating material that can adhere to safety regulations of the food processing industry, or the material that possesses chemical resistance necessary for separating petroleum refinery oily wastewater can be fabricated. The control over filtration fluid will help to overcome efficiency limitations imposed by the oil properties. For example, the water-removing filter can be used for separation of high viscosity oil from mixtures without affecting the permeation flux and separation efficiency; and both oil- and water-removing filters can help to simplify the removal of oils with different densities, which will either float or sink underwater^27^,^44^. Moreover, depending on the technical requirements such as fluid recovery after separation, fouling prevention and material recycling, the suitable separation technique (filtration or absorption) can be selected^64^.

In conclusion, we presented an artificial nanofur surface, which similar to the multifunctional surfaces found in nature, can be adapted to perform different types of oil/water separation: selective separation of either oil or water, variable oil absorption and continuous filtration. The nanofur is fabricated from various polymers, including recyclable and biodegradable, using a scalable hot pulling technique in which nano- and microhairs are created on the polymer surface during polymer separation from the heated sandblasted steel plate. The polymeric nanofur is superhydrophobic and superoleophilic, and can efficiently absorb oil from oil/water mixtures and be reused after washing without losing its wetting properties. The nanofur surfaces made from different polymers vary in hair length and topography, resulting in different oil absorption capacities. The hair morphology of the nanofur can be further controlled by adjusting fabrication parameters such as roughness of the mold. Selective separation of either water or oil from the oil/water mixtures is achieved by reversing the wetting properties of the superhydrophobic nanofur (used for oil removal) to underwater superoleophobic (used for water removal) by argon plasma treatment. The hydrophobic recovery of the underwater superoleophobic plasma-treated nanofur is prevented by storing it in water. Porous nanofur filters fabricated by perforation with microneedles allow continuous gravity driven separation of both fluids from the oil/water mixtures by filtration. Using the described set of simple fabrication and modification techniques a custom oil/water separation platform in terms of material, filtration fluid and separation type can be created, thus, achieving more efficient and environmentally friendly oil/water separation.

## Methods

### Superhydrophobic nanofur fabrication

A hot embossing machine (Jenoptik, Germany) is used to fabricated nanofur by hot pulling. The mold insert is fabricated by sandblasting a steel plate with rough aluminum silicate abrasive, and then with finer aluminum oxide sand with size 53 ± 3 μm, 29 ± 12 μm or 17 ± 1 μm (Hasenfratz Sandstrahltechnik GmbH, Germany). To fabricate polycarbonate (PC) nanofur, the sandblasted plate is heated in the hot embossing machine above the glass transition temperature of PC (*T*_*g*_ = 144 °C), and pressed into the flat PC foil (thickness = 0.2–1 mm) attached to the bottom plate with *T* = 215 °C, embossing velocity 0.4 mm/min, penetration depth 200 μm. Subsequent mold retraction (0.3 mm/min) results in formation of dense nano- and microhairs. Samples used for oil/water filtration and hydrophobic recovery experiments were fabricated using a modified hot pulling technique in which initial PC films were attached to a sacrificial cyclo-olefin-copolymer layer^51^. Polymers used for nanofur fabrication include PC (Makrolon LED2045, Bayer, Germany); PLA (Uhde Inventa-Fischer GmbH, Germany); PET films (Hanita Coatings, Israel); PP, LDPE, HPDE films (Infiana, Germany).

### Characterization and modification

The wetting behavior of the nanofur and hydrophobic recovery of a plasma-treated nanofur (p-nanofur) are characterized by contact angle measurements (OCA 40, DataPhysics Instruments GmbH, Germany), in which 3–4 μl deionized water droplets were dispensed on the surfaces. To measure underwater oil contact angles, 7 μl oil droplet (Total Azolla ZS 10) was dispensed on the p-nanofur placed upside down in a container filled with DI water^54^. Argon plasma treatment of the nanofur surface (0.2 mbar, 30 W, 120 s) was carried out using a reactive ion etching system (Sentech, GmbH, Germany). Porous nanofur was fabricated by perforating nanofur surface with microneedles. Needles with diameter of 160 μm and 25 mm length (Phoenix Medical Ltd., United Kingdom) were manually pushed into nanofur surface placed on a soft spacer material to guarantee puncturing and to minimize damage to the needles. Scanning electron microscopy (SEM) images were taken using Supra 55P microscope (Carl Zeiss, Germany). The nanofur and p-nanofur surface chemistry was investigated by X-ray photoelectron spectroscopy (XPS) using a K-Alpha XPS spectrometer (ThermoFisher Scientific, UK). Data acquisition and processing using the Thermo Avantage software are described elsewhere^65^,^66^. All spectra were referenced to the C 1s peak (C-C, C-H) at 285.0 eV binding energy^61^.

## Additional Information

**How to cite this article**: Kavalenka, M. N. *et al*. Adaptable bioinspired special wetting surface for multifunctional oil/water separation. *Sci. Rep.* **7**, 39970; doi: 10.1038/srep39970 (2017).

**Publisher's note:** Springer Nature remains neutral with regard to jurisdictional claims in published maps and institutional affiliations.

## Figures and Tables

**Figure 1:**
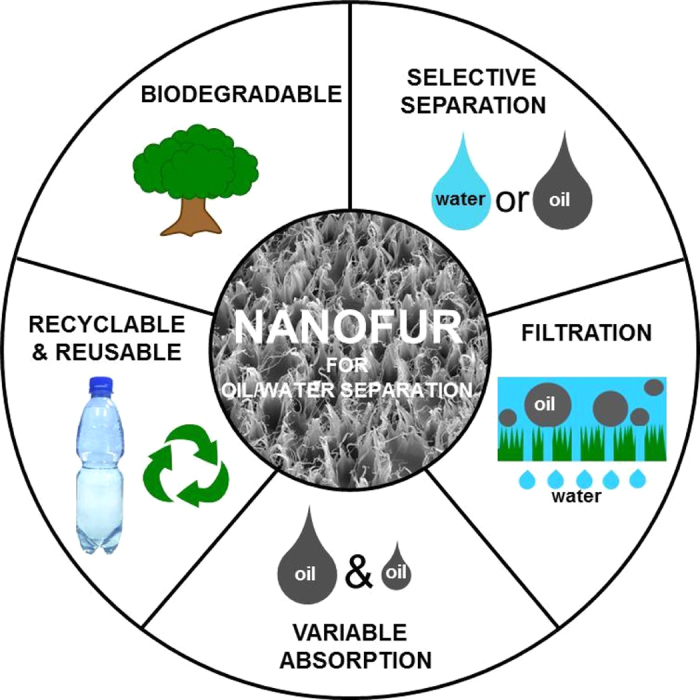
Bioinspired nanofur adaptable for oil/water separation with different functionalities: selective separation of oil or water, variable oil absorption, continuous filtration of oil/water mixtures using porous nanofur. Nanofur can be made from various thermoplastic polymers including biodegradable and recyclable, and can be reused for separation after washing.
